# Analysis of the utility of transbronchial lung biopsy culture under endobronchial ultrasonography with a guide-sheath

**DOI:** 10.1038/s41598-023-43078-x

**Published:** 2023-09-26

**Authors:** Masafumi Shimoda, Kozo Morimoto, Yoshiaki Tanaka, Masashi Ito, Iori Moue, Kozo Yoshimori, Ken Ohta

**Affiliations:** 1 Respiratory Disease Center, Fukujuji Hospital, Japan Anti-Tuberculosis Association (JATA), 3–1–24 Mastuyama, Kiyose, Tokyo 204–8522 Japan; 2 Division of Clinical Research, Fukujuji Hospital, Japan Anti-Tuberculosis Association, Kiyose, Tokyo Japan; 3 Department of Clinical Mycobacteriosis, Nagasaki University Graduate School of Biomedical Sciences, Nagasaki, Japan

**Keywords:** Infectious diseases, Respiratory tract diseases

## Abstract

Transbronchial lung biopsy (TBLB) culture is not common in clinical practice, and TBLB culture for patients with mycobacterial disease provide limited value because the diagnostic accuracy of TBLB culture is very low. Recently, bronchoscopic devices have been further developed, such as endobronchial ultrasonography with a guide-sheath (EBUS-GS). Therefore, this study investigated the utility of TBLB culture obtained by using EBUS-GS compared to washing cultures. A total of 31 patients who underwent TBLB culture by using EBUS-GS (GS-TBLB) were collected retrospectively at Fukujuji Hospital from January 2018 to December 2022. The diagnostic accuracies of GS-TBLB culture and bronchial and device washing cultures (namely, washing culture) were compared. The patients comprised 13 individuals with nontuberculous mycobacteriosis, 7 with pulmonary aspergillosis, 6 with lung abscess, and 5 with pulmonary tuberculosis. The diagnostic accuracy of GS-TBLB culture was lower to that of TBLB culture than those of washing culture (n = 11 [35.5%] vs. n = 20 [64.5%], *p* = 0.016), and there was only one patient with positive GS-TBLB culture results and negative washing culture results. Comparing between patients with mycobacteria and non-mycobacteria, GS-TBLB culture positivity were no significant difference between patients with mycobacteria and non-mycobacteria (n = 6 [33.3%] vs. n = 5 [38.5%], *p* = 1.000), however, patients with mycobacteria diagnosed by washing culture more than those with non-mycobacteria (n = 15 [83.3%] vs. n = 5 [38.5%], *p* = 0.021). Our results demonstrate that the utility of TBLB culture for the diagnosis of pulmonary infections might provide limited value even if EBUS-GS is performed and lung tissue is successfully obtained.

## Introduction

Transbronchial lung biopsy (TBLB) is commonly performed for histopathological examinations, but the use of TBLB culture is not widespread and has been the subject of limited studies^1^. The diagnostic accuracy of TBLB culture has been shown to be 4.1–42% for patients with mycobacterial disease, including pulmonary tuberculosis (TB) and nontuberculous mycobacteriosis (NTM), and TBLB culture adds little to the results obtained by culturing bronchial washings along with culturing prebronchoscopy and postbronchoscopy sputum^2,3^. Recently, bronchoscopic devices have been further developed, such as endobronchial ultrasonography (EBUS) with a guide-sheath (EBUS-GS), but previous reports did not include these advanced devices. EBUS-GS is a useful method for collecting samples^4^, and a previous report demonstrated that TBLB culture obtained by EBUS-GS can help to diagnose tuberculosis and fungal and bacterial infections^1^. However, comparison of the diagnostic accuracy between TBLB culture and bronchial and device washing cultures (namely, washing culture) has not been performed. Therefore, this study investigated the utility of TBLB culture obtained by using EBUS-GS (GS-TBLB) compared to that obtained with washing cultures.

## Materials and methods

### Study design and setting

A total of 84 patients who underwent TBLB culture by bronchoscopy were collected retrospectively at Fukujuji Hospital from January 2018 to December 2022. Thirty-nine patients who ultimately diagnosed with conditions other than infectious diseases, eleven patients with no EBUS-GS usage, two patients with NTM without acid-fast bacilli (AFB) culture from TBLB, and one patient with pulmonary TB who underwent bronchoscopy after receiving anti-TB treatment were excluded. Finally, thirty-one patients were reviewed, including 13 patients with NTM, 7 patients with pulmonary aspergillosis, 6 patients with lung abscess, and 5 patients with pulmonary TB. All patients underwent TBLB, brushings, and bronchial washings during bronchoscopy. TBLB and brushings were performed through EBUS-GS. The device washings included brushings, forceps, and guide-sheath washings in saline, and the washing cultures were examined through both bronchial washing and device washing combined. The diagnostic accuracy of GS-TBLB culture and washing culture were compared.

### Definition

Pulmonary NTM was diagnosed according to the diagnostic criteria in the guidelines: positive culture results from at least two separate expectorated sputum samples, positive culture results from at least one bronchial wash or lung biopsy, or transbronchial lung biopsy with mycobacterial histologic features (granulomatous inflammation or AFB)^5^. Pulmonary TB was diagnosed according to the presence of *M. tuberculosis* in the culture of any specimen and/or positive transcription–reverse-transcription concerted reaction (TRC) for TB results, which is a type of nucleic acid amplification test. Pulmonary aspergillosis was diagnosed in patients with clinically and radiologically abnormal sites consistent with *Aspergillus* infection based on the presence of *Aspergillus* specimens in the culture of any specimen, hyphae with evidence of associated tissue damage on histopathological/cytopathological examination, or positivity for *Aspergillus* antigen/antibody^6^. Patients with lung abscess diagnosed for the lesion of lung abscess improved by antibiotics therapy with no evidence of alternate causes.

The size of the target lesion was assessed using computed tomography scans that were conducted within 1 month prior to bronchoscopy. Among the patients, 8 had a mass as the target lesion, 19 had nodules, and 4 had consolidation. The size of the target lesion was determined by measuring the tumor diameter for masses/nodules and the maximum distance within the area with consolidation shadows for consolidations.

### TBLB culture procedure

Bronchoscopy was conducted using pharyngeal anesthesia with a 2% xylocaine solution, along with intravenous premedication. The premedication included 1–4 mg of midazolam as a sedative and/or 17.5–35 mg of pethidine as an analgesic. The bronchoscope was inserted transorally, with a 2% xylocaine solution applied through the instrumentation channel of the bronchoscope. Prior to biopsy, an ultrasound scanner (UM-S20-17S, Olympus Inc, Japan) was employed to visualize the position, size, and shape of the lesion in order to plan the puncture. TBLB and brushings were performed through the GS (single use guide sheath kit K-401, Olympus Inc, Japan), with 1–3 samples of TBLB was placed in saline for culture and sent to the microbiology laboratory in our hospital. The specimen was crushed on a glass slide and then smeared and cultured. Specimen processing was promptly carried out upon arrival at the laboratory, although if storage was required, it was kept at 4 °C. The biopsy specimens other than TBLB culture sent to histopathological examination. The specimen processing was promptly performed upon arrival at the laboratory, but when storage was necessary, it was kept at 4 °C. Bronchial washing, without the GS, was performed using 20 mL of saline. Bronchoalveolar lavage was not conducted for any of the patients.

Microbe detection methods comprised acid-fast staining, standard bacterial culture, regular fungal culture, mycobacterium culture, and TRC for TB.

### Ethical approval

The study was approved by the Institutional Review Board of Fukujuji Hospital. The need for Inform consent was waived by Institutional Review Board of Fukujuji Hospital (Study number: 21041). All methods were performed in accordance with the Declaration of Helsinki.

### Statistical methods

All data were analysed and processed using EZR, version 1.53^7^. McNemar's chi-squared test were used for the diagnostic accuracies of GS-TBLB culture or/and GS-TBLB histopathology and washing culture. Pearson’s chi-squared test were used for comparisons between patients with mycobacteria and non-mycobacteria. The Cochran–Armitage trend test was used to compare between TBLB culture positivity and the number of GS-TBLB for culture. The level of statistical significance was set at *p* = 0.05 (2-tailed).

## Result

The median age of all patients was 62.0 years (interquartile range (IQR) 42.5–70.5), and there were 18 males (58.1%). The positive rates of GS-TBLB culture were 35.5% (n = 11); including 16.1% (n = 5) in the patients with NTM, 3.2% (n = 1) in the patients with TB, 6.5% (n = 2) in the patients with pulmonary aspergillosis, 6.5% (n = 2) in the patients with lung abscess, and 3.2% (n = 1) patient with pneumonia (*Pseudomonas aeruginosa*). The diagnostic accuracy of GS-TBLB culture was significantly lower than that of washing culture (35.5% [n = 11] vs. 64.5% [n = 20],* p* = 0.016) (Table 1), and there was only one patient with positive GS-TBLB culture results and negative washing culture results. Even among the 25 patients with inflammatory lesions on GS-TBLB histopathology, the diagnostic accuracy of GS-TBLB culture was 40.0% (n = 10). GS-TBLB histopathology in 16 patients showed epithelioid cell granulomas, fungus, or abscess, which indicated infection of mycobacterium, fungus, or lung abscess. The diagnostic accuracy of combined GS-TBLB culture and histopathological findings was no significant difference comparing to that of washing culture (*p* = 1.000), 7 of 16 patients had positive results for both GS-TBLB culture and histopathology combined, and 4 patients with negative washing culture results could diagnosed by GS-TBLB culture and/or histopathological findings. There was no significant difference between the diagnostic accuracy of GS-TBLB culture and those of GS-TBLB histopathology (35.5% [n = 11] vs. 51.6% [n = 16], *p* = 0.267). Four patients with negative washing culture results could diagnosed by GS-TBLB culture and/or histopathological findings. The size of target lesion showed no significant difference between patients with positive GS-TBLB culture results and those with negative GS-TBLB culture results (median 23.7 mm [IQR 18.0–39.9] vs. 20.7 mm [13.1–31.9], *p* = 0.427). The number of GS-TBLB cultures performed was once in 26 patients, twice in 3 patients, and three times in 2 patients. There was no significant difference between the number of GS-TBLB obtained and positive culture results for GS-TBLB (The Cochran–Armitage trend test *p* = 0.725).

**Table 1:** Comparison of positive results between TBLB culture and washing culture based on McNemar's chi-squared test.

	Washing culture		*p* value
	Positive	Negative	
TBLB culture			
Positive	10	1	0.016
Negative	10	10	
TBLB histopathological findings*			
Positive	13	3	0.343
Negative	7	8	
TBLB culture and histopathological findings			
Positive	16	4	1.000
Negative	4	7	

*Positive histopathological findings showed presence of epithelioid cell granulomas, fungus, or abscess, which indicated infection of mycobacterium, fungus, or lung abscess.

TBLB, transbronchial lung biopsy.

Next, comparisons between patients with mycobacteria and non-mycobacteria were shown in Table 2. GS-TBLB culture positivity were no significant difference between patients with mycobacteria and non-mycobacteria (n = 6 [33.3%] vs. n = 5 [38.5%], *p* = 1.000). Conversely, patients with mycobacteria diagnosed by washing culture more than those with non-mycobacteria (n = 15 [83.3%] vs. n = 5 [38.5%], *p* = 0.021).

**Table 2:** Comparison of TBLB/washing culture positivity and mycobacteria or non-mycobacteria.

	Mycobacteria	Non-mycobacteria	*p value*
TBLB culture			
Positive	6	5	1.000
Negative	12	8	
Washing culture			
Positive	15	5	0.021
Negative	3	8	

TBLB, transbronchial lung biopsy.

Seven patients (22.6%) had complications after bronchoscopy, such as fever requiring new antibiotic treatment (n = 2), lung abscess (n = 2), haemoptysis for 3 days or more (n = 2), vomiting (n = 1), and pneumothorax (n = 1).

## Discussion

Our results demonstrate that the utility of GS-TBLB culture for the diagnosis of pulmonary infections provided limited value, even when using EBUS-GS technique. Although EBUS-GS could help diagnose tuberculosis, fungal, and bacterial infections^1^, the diagnostic accuracy of GS-TBLB culture in our data were similar to those of previous reports regardless of whether EBUS-GS was used or not^2,3^, and only one patient was diagnosed solely by GS-TBLB culture. The GS-TBLB procedure was successful in many patients, as histopathological findings showed presence of epithelioid cell granulomas, fungus, or abscess. However, the combination of GS-TBLB culture and histopathology was not superior to washing culture regarding diagnostic accuracy. Therefore, even if EBUS-GS is performed and lung tissue is successfully obtained, the utility of GS-TBLB culture is low. For localized lesions of infection, GS-TBLB biopsy can reach the lesion^4^. Brushing through GS can also approach the lesion similar to GS-TBLB; therefore, device washing of brushing and bronchial washing after brushing are likely to culture causative pathogens. Furthermore, a previous report exhibits that GS-TBLB culture is limited utility for peripheral lung lesion. In addition to this evidence, our study further confirmed its lack of effectiveness regardless of the size of the target lesion. On the other hand, culturing non-mycobacteria from washing specimens was more challenging compared to culturing mycobacteria from the same type of specimens, and positive culture ratio of non-mycobacteria was similar between GS-TBLB and washing specimen. The patient with positive GS-TBLB culture results and negative washing culture results was diagnosed with lung abscess. This suggests that performing TBLB culture might be considered in patients suspected of having non-mycobacterial infection by using EBUS-GS because identification of pathogens from GS-TBLB culture can also aid in the selection of appropriate the selection of antibiotics^1^.

Although a previous report shows that some cases of patients who underwent GS-TBLB for pathogens other than mycobacteria were found to have successfully identified causative pathogens from GS-TBLB culture^1^, no report has investigated a comparison of the utility of GS-TBLB culture between patients with mycobacterial and non-mycobacterial infection. Generally, TBLB culture is not considered useful for diagnosing mycobacteria, including TB and mycobacterium avium complex^2,3^. In sputum-negative TB patients, there is no difference in the diagnostic rate between combined BAL and TBLB histology and combined BAL, TBLB histology, and TBLB culture^8^. All MAC patients who exhibited positive TBLB findings also showed positive acid fast bacilli in smear or/and culture^9^. On the other hand, a previous report has indicated that the use of GeneXpert in bronchoalveolar lavage may eliminate the need for TBLB in increasing the diagnostic yield for TB^10^, and it has been reported that metagenomic next-generation sequencing (mNGS) of TBLB specimens is effective for the diagnosis of infectious peripheral pulmonary lesions; therefore, it may be worth considering TBLB if mNGS is available^11^.

GS-TBLB might lead to the development of a postbronchial respiratory infection^12^. In a previous study, no cases of postbronchoscopic respiratory infections were reported in patients who underwent GS-TBLB culture^1^, however, in our study, 6.5% of patients developed lung abscess after bronchoscopy. The incidence of developing lung abscesses after bronchoscopy has increased in recent years, which may be attributed to the advancement in bronchoscopic techniques, such as EBUS-GS^12^. Furthermore, pneumothorax also developed after bronchoscopy in both our study and a previous study^1^. Therefore, clinicians should note the complications of using EBUS-GS under bronchoscopy. Patients who do not require histopathological examinations might not need a TBLB culture.

This investigation had several limitations. The study was conducted retrospectively in a single center. The sample size was small, and various pathogens were included. Patients who underwent GS-TBLB culture were selected by the clinician; therefore, patient backgrounds were variable.

## Conclusion

Our results demonstrate that the utility of TBLB culture for the diagnosis of pulmonary infections might provide limited value even if EBUS-GS is performed and lung tissue is successfully obtained.

## Data Availability

The datasets used and/or analysed during the current study are available from the corresponding author on reasonable request.
